# Modified matrix solid phase dispersion-HPLC method for determination of pesticide residue in vegetables and their impact on human health: A risk assessment

**DOI:** 10.3389/fchem.2022.1084350

**Published:** 2022-12-08

**Authors:** Majida Mujahid, Shoomaila Latif, Mahmood Ahmed, Warda Shehzadi, Muhammad Imran, Muhammad Ahmad, Asnuzilawati Asari, Muhammad Jehangir, Zaid Mahmud

**Affiliations:** ^1^ Department of Chemistry, The University of Lahore, Lahore, Pakistan; ^2^ School of Physical Sciences, University of the Punjab, Lahore, Pakistan; ^3^ Department of Chemistry, Division of Science and Technology, University of Education, Lahore, Pakistan; ^4^ Centre for Inorganic Chemistry, School of Chemistry, University of the Punjab, Lahore, Pakistan; ^5^ Faculty of Science and Marine Environment, Universiti Malaysia Terengganu, Kuala Nerus, Terengganu, Malaysia; ^6^ Novamed Pharmaceuticals (Pvt) Limited, Lahore, Pakistan; ^7^ The Department of Chemistry, FC College (A Chartered University), Lahore, Pakistan

**Keywords:** fungicides, insecticides, extraction, estimated daily intake, target hazard quotient

## Abstract

The use of pesticides is unavoidable in agricultural practices. This class of chemicals is highly toxic for the environment as well as for humans. The present work was carried out to assess the presence of some pesticides (diafenthiuron, lufenuron, azoxystrobin, difenoconazole, and chlorothalonil) residues in five of the very commonly used vegetables (eggplant, capsicum, apple gourd, cauliflower, and sponge gourd). Matrix solid phase dispersion (MSPD) technique was used to extract the pesticides and subsequently their quantification was performed through high performance liquid chromatography (HPLC) coupled to ultraviolet-visible (UV-Vis) detector. The elution was accomplished at wavelength of 254 nm by injecting 20 µL of standards or samples into chromatographic system. The mobile phase consisted of acetonitrile and water (80:20 v/v), where the flow rate was adjusted at 1.0 ml/min. The linearity was good (*R* ^2^ ≥ 0.994) over a concentration range from 20 to 100 μg/ml for the investigated pesticides. The low detection limits showed a quite appreciable potential of the method to detect (1.12–1.61 μg/L) and quantify (3.73–5.36 μg/ml) the pesticides under study. The accuracy was demonstrated in terms of percent recovery which ranged between 88.5% and 116.9% for all the pesticides under investigation. These results justify the suitability of the technique for the intended purpose. The concentration of difenoconazole in apple gourd (20.97 mg/kg), cauliflower (10.28 mg/kg), and sponge gourd (40.32 mg/kg) whereas diafenthiuron in cauliflower (0.66 mg/kg) exceeded the maximum residue level (MRLs) as defined by Food and Agriculture Organization of the United Nations and the World Health Organization (FAO/WHO). Target hazard quotient (THQ) values of difenoconazole and diafenthiuron (except for adults) were more than one which indicates the significant effect on human health on consumption of apple gourd, cauliflower, and sponge gourd.

## 1 Introduction

Vegetables are a rich source of various nutrients such as fibers, carbohydrates, vitamins, minerals, and different polyphenols. By virtue of containing a variety of important natural ingredients, human beings use vegetables for the life sustainability as an essential component of their food. Several studies show that a regular use of vegetables alleviates the risk of various diseases including different types of cancers, diabetes, heart disease, stroke, high blood pressure, and some chronic diseases as well (Yang et al., 2009; Hu et al., 2013; Ul-Haq et al., 2021). Pesticides are a prime group of chemical and their use is unavoidable in agricultural practices. However, an excessive use of this class of chemicals can produce a toxic effect on the environment as well as on the human health. Severe infestation of pests, especially during initial stages of a crop life, can potentially reduce the crop yields (Kim et al., 2013; Wright et al., 2013; Machado et al., 2016; Mudge et al., 2016). Therefore, agricultural sector would suffer from huge economic losses if pesticides are not applied when required. The use of pesticides significantly increases crop yields and thus the financial margins. Pesticides such as fungicides, insecticides, herbicides, and rodenticides are frequently being applied in the agricultural sector to minimize the damage to vegetables caused by certain insects, pests, fungi, and weeds. But their excessive use becomes hazardous as these harmful chemicals can be retained in vegetables as a residue. Consequently, these pesticides tend to accumulate in the fat tissues of humans after consumption (Nakata et al., 2002; Syed et al., 2014).

Despite the benefits of increasing the yields by protecting the crops from harmful pests, the dose of pesticides must not exceed the limits provided by the pest control board. A large part of the pesticides’ impact on the environment occurs at the time of their application, however a large portion of them can move or float off-site thus bringing poisonous impacts to the environment and humans. The dynamics of different pesticides depend on their application to a particular specie under investigation (Stephen et al., 2017). The applied pesticides are delivered promptly into the environment, however the portions arriving at the proposed target and unintended targets can fluctuate considerably. Thus these hazardous chemicals may enter the environment, accumulate in the food chain, and cause toxic effects to humans (Akoto et al., 2013). In contrast to most drugs, pesticides are generally applied to kill more than one pest species having different degrees of sensitivity. Therefore, the recommended dose should kill the most tolerant pest species. Otherwise, a heavy application of pesticides can cause a diffuse contamination to the environment as well as can result in a widespread pest resistance leading to a disturbance in the natural balance, and hazards to humans, and wildlife (Stephen et al., 2017).

Several analytical procedures followed by various extraction techniques including gel permeation chromatographic system (GPC) with a graphitized carbon columns (Ueno et al., 2004; Wu et al., 2016), gas chromatographic (GC) method of analysis with electron captured (EC) and mass spectrometric (MS) detector with prior extraction by various methods like supercritical fluid (SF), liquid-liquid extraction (LLE), solid phase extraction (SPE), magnetic solid-phase extraction (MSPE), solid-phase microextraction (SPME), stir bar sorptive extraction (SBSE), hollow-fiber liquid-phase microextraction (HF-LMPE), and dispersive liquid-liquid microextraction (DLLME) (Kaihara et al., 2002; Anastassiades et al., 2003; Diez et al., 2006; Rai et al., 2016; Lee et al., 2017; Narenderan et al., 2019) have been reported for the quantification of pesticide residues in agricultural products including vegetables and fruits.

The above mentioned sample preparation methods have some disadvantages linked to them. For example, the disadvantages of LLE are low selectivity along-with high consumption of organic solvents which accounts for production of toxic vapors, time consuming due to multi-step extraction process (Musarurwa and Tavengwa, 2021). SPE, MSPE, and SPME involve poor reproducibility of isolation and enrichment, higher cost, sorbent media, and clogging/breaking of sorbent bed (Farooq et al., 2022; Yeganeh et al., 2022; Zhang et al., 2022). The drawbacks of SBSE are difficulties in removing the stir-bar from the samples and its rinsing, and desorption requires several steps (Veloo and Ibrahim, 2021). HF-LPME needs reconditioning of membrane as well as longer extraction times (Zuluaga et al., 2021). Finally, DLLME requires use of three solvents with limited choice of solvents, and is considered not suitable for a sample having complex matrix composition due to which it shows less selectivity with low precision (Nasiri et al., 2020; Khosrowshahi et al., 2021).

**FIGURE 1:**
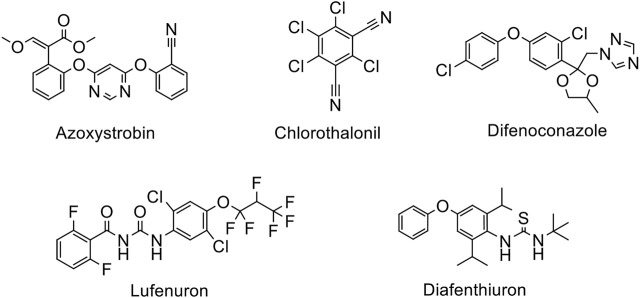
Structures of the pesticides studied.

In the current study, an attempt was made to resolve the problem associated with the mentioned techniques, where the extraction was carried out by a simple and effective procedure i.e. modified matrix solid phase dispersion (MSPD). This technique is adaptable, simple, fast due to shorter sample treatment times, and convenient in handling as compared to the conventional methods (Nasiri et al., 2020). MSPD is developed on the basis of sorbent material dispersion into a sample solution which contains an analyte. An appropriate solvent is employed for the analyte desorption after the adsorption process. Besides, conditioning and washing steps are not required for MSPD technique, which makes it simpler and faster as compared to other solid phase extraction methods. Subsequently, high performance liquid chromatography (HPLC) coupled to ultraviolet/visible detector (HPLC/UV-Vis) was employed for the simultaneous quantification of five pesticides (diafenthiuron, lufenuron, azoxystrobin, difenoconazole, and chlorothalonil) in vegetables (eggplant, capsicum, apple gourd, cauliflower, sponge gourd) which are commonly employed for the protection of crops against different pests (Figure 1). The main objective of this study was to assess the presence of some pesticides’ residues present in selected vegetable samples. The results were compared with the permitted limits provided by the regulatory authority. Besides, we evaluated whether the outcomes followed existing guidelines especially the ones given by FAO/WHO. At long last, we also considered the appropriateness of the studied commodities for human consumption keeping in view the official MRLs (maximum residue levels). Assessment of human health risk by exposure to vegetable contaminated with pesticides was also evaluated by determining the value of estimated daily intake (EDI, in mg/kg/day) and non-carcinogenic target hazard quotient (THQ).

## 2 Experimental

### 2.1 Reagents and solutions

Diafenthiuron, lufenuron, azoxystrobin, difenoconazole, and chlorothalonil (Sigma Aldrich, United States), sodium sulphate (Merck, Darmstadt, Germany), florisil (60–100 mesh, Sigma Aldrich, United States) of analytical reagent grade, and HPLC grade acetonitrile (Panreac, Barcelona, Spain) were acquired from Hajvery Scientific Store, Lahore-Pakistan. The GenPure (Thermo Scientific, United States) water system was used to prepare ultrapure water (18 MΩ cm resistivity). Pesticide standard stock solutions were prepared in mobile phase (acetonitrile: water, 80:20 v/v) through which further dilution was made to prepare the working standard solutions of each pesticide. The solutions were stored at 4°C until their use. Calibration standard solutions were prepared in the range from 20 to 100 μg/ml (20 μg/ml, 40 μg/ml, 60 μg/ml, 80 μg/ml, and 100 μg/ml).

### 2.2 Sample collection and pre-treatment

The vegetable samples which were grown in the dedicated land for experimental purposes were provided by Plant Pathology Laboratory, Agriculture Department, University of the Punjab, Lahore-Pakistan. Pesticide-free vegetables controlled by the Residue Control Laboratory, University of the Punjab, Lahore-Pakistan were used as blank, whereas the blank samples were spiked with respective pesticides for validation studies. To guarantee the maximum sample extraction efficiency and get accurate results, 1 kg of each vegetable (eggplant, capsicum, apple gourd, cauliflower, sponge gourd) was collected. Each vegetable was lyophilized and chopped into small pieces which were kept in the dark at −20°C until further analysis. The plant used in this research comply the criteria and policy established by “Convention on the Trade in Endangered Species of Wild Fauna and Flora and the IUCN Policy Statement on Research Involving Species at Risk of Extinction.

### 2.3 Sample extraction

#### 2.3.1 Solid phase extraction

**FIGURE 2:**
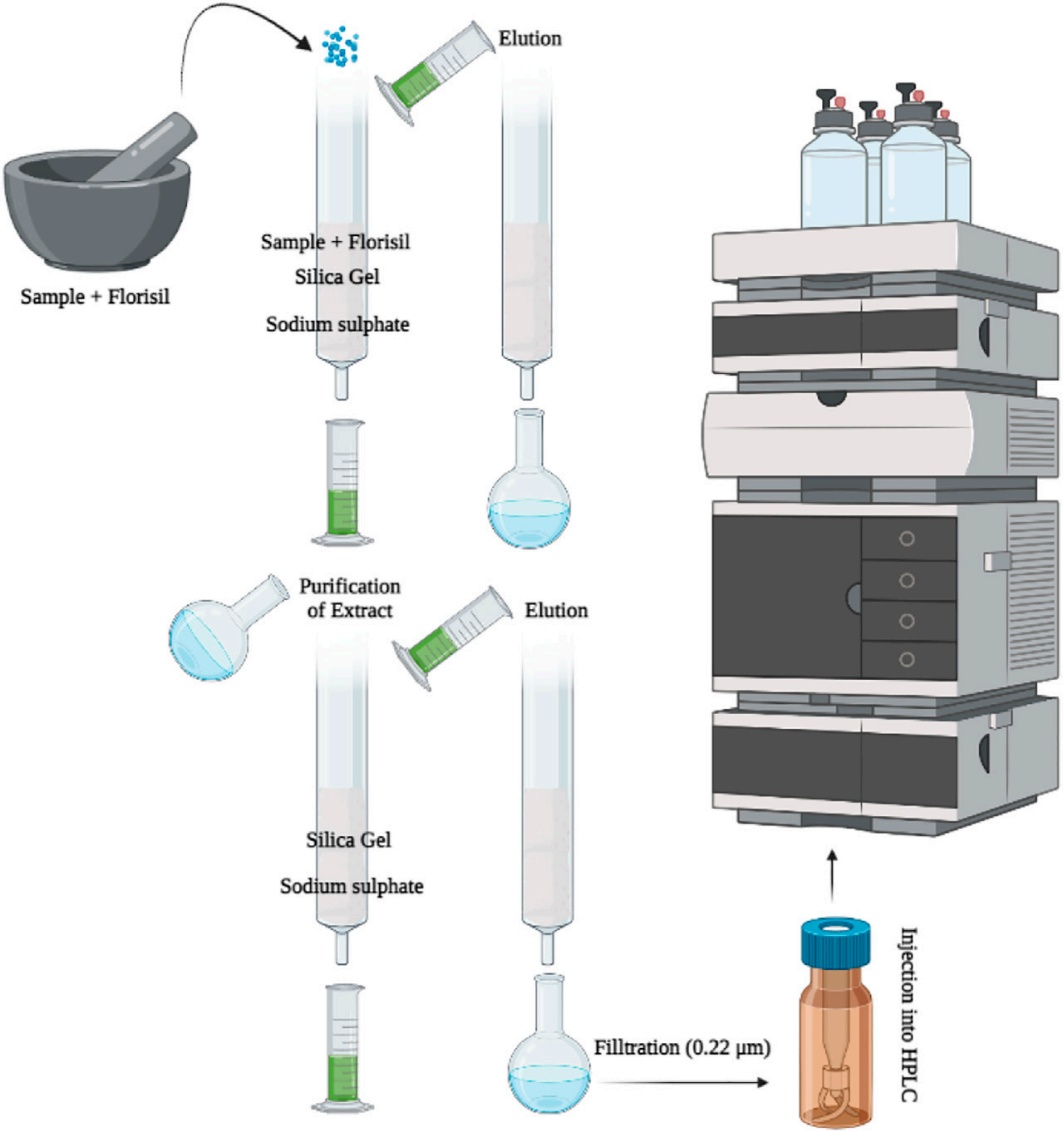
Extraction of pesticide residues in vegetables by MSPD and quantification by HPLC.

The MSPD (Figure 2) method was opted for the extraction of all pesticide residue from respective vegetables under investigation. In the MSPD technique, florisil (4.0 g) which was previously activated at 600°C was used as dispersing sorbent, whereas the elution of analytes was achieved by acetonitrile. 4.0 g of each vegetable sample separately and solid support-florisil were triturated with the help of mortar and pestle containing acetonitrile to get the homogeneous mixture. The glass column (400 mm × 15 mm) was packed with 2.5 g silica gel and 5.0 g anhydrous sodium sulphate. The previously homogenized sample was transferred to packed column and elution was performed with acetonitrile (15.0 ml, HPLC grade). Mostly, acetone and ethyl acetate are selected for extraction clean-up in SPE methods but these solvents did not work efficiently in MSPD, therefore acetonitrile was selected as the elution solvent for the extraction purpose in the current studies (Capriotti et al., 2015).

The eluate of each vegetable was collected in a round bottom evaporating flask and then volume was reduced to approximately 2.0 ml using a rotary evaporator at 40°C. The obtained eluate was diluted further with 2.0 ml of acetonitrile to get the final aliquot for analysis by HPLC.

### 2.4 Chromatography

A liquid chromatographic system of Agilent 1,260 (Infinity II LC System) equipped with UV-Vis detector (G7114 A), and isocratic pump (G7110 B) was used to perform chromatography. The separation was accomplished at room temperature through C-18 column (150 mm × 4.6 mm, 5 μm). The mobile phase comprising of acetonitrile: water (80:20 v/v) mixture was pumped through the LC system. The flow rate was set at 1.0 ml/min. A 20.0 µL of sample was injected and the detection was performed at 254 nm. A nylon membrane filter (0.22 µm, Sartorius, Germany) was used to filter the mobile phase, working standards and sample solutions prior to injection into LC system whereas data was acquired by Open Lab CDS software.

### 2.5 Method validation

European Union guidelines (Document Nº SANTE/12682/2019) (Commission, 2019) were followed to validate the authenticity of proposed method for the determination of pesticide residue in vegetable samples. To establish the validity of the suggested method, different parameters such as matrix effect, linearity, accuracy, precision, limit of detection (LOD), and limit of quantitation (LOQ) were considered and measured. Selectivity is referred to a successful elution of an analyte of interest from a complex mixture in the presence of some other substance, whereas linearity is referred to responsiveness of an instrument to a particular specie within a specific concentration range. Linearity (y = mx + c) shows the ability of the method to verify that response of analyte is directly proportional to a specific concentration range. For selected concentration range of each analyte, important parameters such as slope (m), intercept (c), and coefficient of determination (*R* ^2^) have been tabulated. Accuracy measures the proximity between the obtained and true values and implies that there is no inherent systematic error or bias. A bias determines the deviation of obtained value from the true value.

The recovery studies were performed to evaluate the accuracy by spiking the sample with each pesticide at low (20 μg/ml), medium (60 μg/ml), and high (100 μg/ml) concentration level. The precision of the proposed analytical procedure was presented in % relative standard deviation (RSD) which is the closeness of agreement among the individual values attained when the multiple homogenous samples were analysed repeatedly under defined conditions. Repeatability (on the same day by the same operator) and reproducibility (on the same day by the different operator) were performed to assess the precision. The lowest concentration in the calibration curve (20 μg/ml) was spiked to the blank matrix of each vegetable and the matrix effect was reported in percentage recovery and same concentration was spiked with real sample extracts.

Under the appropriate conditions of the chromatographic method, the lowest amount of analyte that is detectable but not necessarily quantitated is the limit of detection (LOD = 3 σ/S) while the amount that is determined with acceptable accuracy and precision is the limit of quantitation (LOQ = 10 σ/S), where *σ* is SD (standard deviation) of interception while S is the slope of the regression curve. Detection limits determined the sensitivity of the method (Ahmed et al., 2013; Ahmad et al., 2016; Qadir et al., 2016; Jehangir et al., 2020; Ahmed et al., 2021).

### 2.6 Human health risk assessment

Assessment of human health risk by exposure to vegetable contaminated with pesticides was evaluated by determining the value of estimated daily intake (EDI, in mg/kg/day) and non-carcinogenic target hazard quotient (THQ). EDI is maximum amount of any chemical to which a human population including sensitive subgroups like children gets exposed after ingesting it daily over a lifetime but without suffering a deleterious effect. EDI of a pesticide including diafenthiuron, lufenuron, azoxystrobin, difenoconazole, and chlorothalonil was calculated by using the formula.

Where IR is the ingestion rate of vegetables which is 0.345 kg/day and 0.232 kg/day for adult and children respectively, C is the concentration (mg/kg) of pesticide residue found in vegetables, BW is the average body weight which is taken 73.0 kg and 32.3 kg for adult and children respectively. THQ is terminology used to characterize the risk associated with amount of chemical in specific food after its exposure and its value is determined by dividing the EDI with RfD (oral reference dose in mg/kg BW/day).

For a value of THQ <1, the exposed population experiences no significant health risk, whereas RfD values of diafenthiuron, lufenuron, azoxystrobin, difenoconazole and, chlorothalonil are 0.003, 0.02, 0.20, 0.01, and 0.015 mg/kg. BW/day (Kumari and John, 2019; APVMA, 2022; Hasnaki et al., 2022) respectively.

## 3 Results and discussion

### 3.1 Chromatography

The final analysis of pesticide residue in vegetable samples was performed after the successful extraction of respective pesticides (azoxystrobin, chlorothalonil, difenoconazole, lufenuron, and diafenthiuron) by MSPD. All the pesticides under investigation were satisfactorily separated and determined with sufficient resolution in the presence of matrix, where matrix effect is vitally important in the development of a liquid chromatographic (LC) method.

**FIGURE 3:**
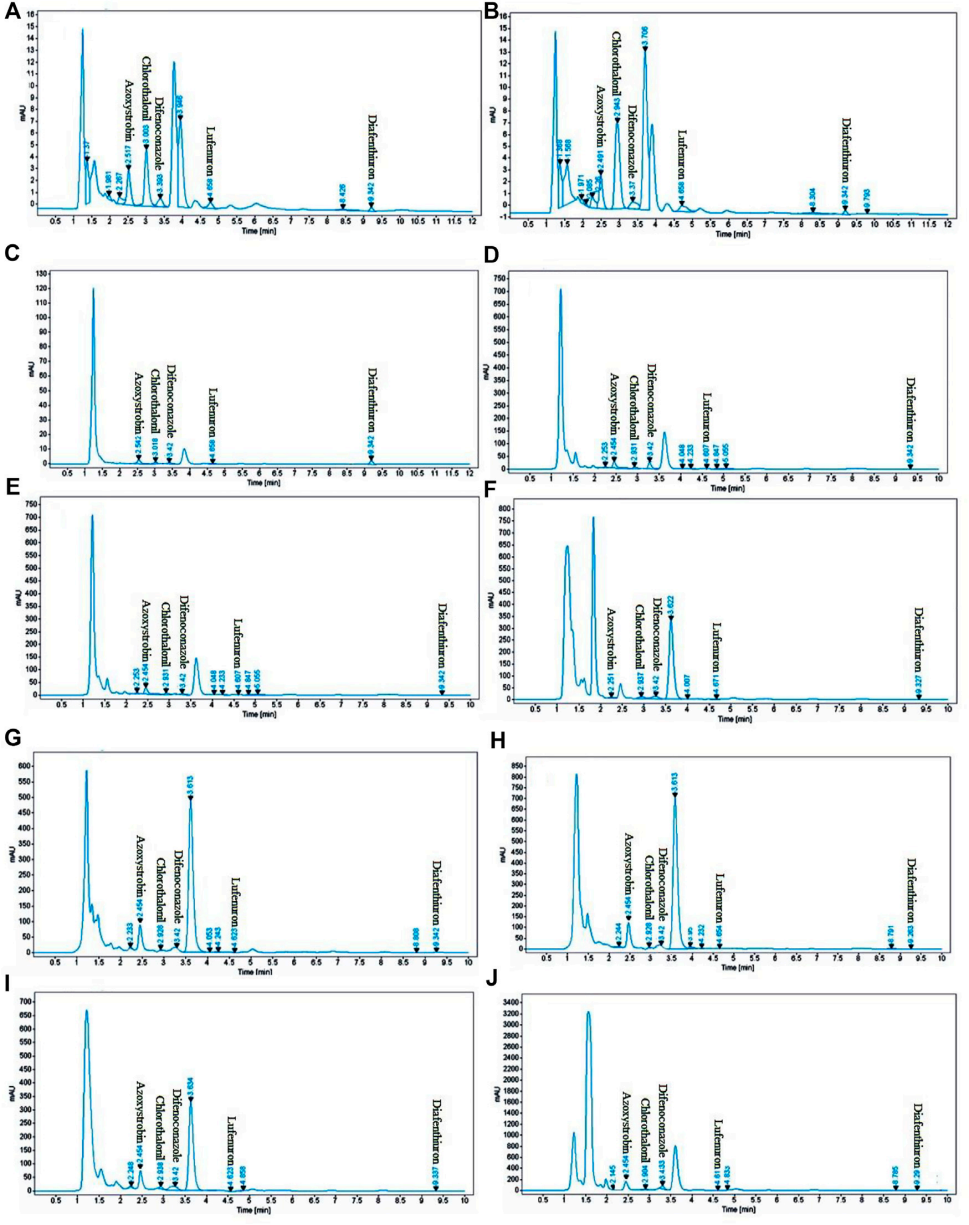
Typical HPLC chromatograms of eggplant [**(A)**: spiked blank as standard, **(B)** sample], capsicum [**(C)**: spiked blank as standard, **(D)** sample], apple gourd [**(E)**: spiked blank as standard, **(F)** sample], cauliflower [**(G)**: spiked blank as standard, **(H)** sample], and sponge gourd [**(I)**: spiked blank as standard, **(J)** sample], spiked concentration. (20 μg/ml).

To determine the absorption maximum (λ_max_), the scanning was performed over the entire range of UV-Vis spectrum (200–800 nm). A λ_max_ of 254 nm was selected because all analytes showed absorption maximum at this wavelength. Identification and quantification of each analyte were performed by retention time and peak areas and by comparing them with the working standards under similar conditions as shown in the chromatogram (Figure 3).

### 3.2 Method validations

The validation assessment of proposed MSPD-HPLC analytical method was performed by taking in account the coefficient of determination (*R* ^2^) from linearity, recovery for accuracy, precision, matrix effect, and detection limits (LOD and LOQ). The selectivity of the proposed method was evaluated by spiking the lowest concentration of regression curve (20 μg/ml) of each pesticide. The selectivity of the method was determined by quantifying each pesticide in a complex sample at its retention time while having no interference of other compounds present in the sample. The linearity of chromatographic methods was evaluated by plotting triplicate five-point (as described in section 2.5) linear plots. The obtained regression curves were linear for all five pesticides (azoxystrobin, chlorothalonil, difenoconazole, lufenuron, and diafenthiuron) under investigation with a coefficient of determination (*R* ^2^) ≥ 0.994 (Table 1).

**TABLE 1:** Parameters of validation studies.

Parameters	Pesticide				
	Azoxystrobin	Chlorothalonil	Difenoconazole	Lufenuron	Diafenthiuron
Concentration range (µg/ml)	20–100	20–100	20–100	20–100	20–100
Regression equation	y = 1.919x + 40.29	y = 0.241x + 59.59	y = 0.297x + 2.89	y = 0.065x + 39.82	y = 0.0158x+38.437
Coefficient of determination (*R* ^2^)	0.996	0.996	0.994	0.996	0.997
Retention time (min.)	2.5	2.9	3.4	4.6	9.3
LOD (μg/ml)	1.61	1.51	1.12	1.21	1.51
LOQ (μg/ml)	5.36	5.03	3.73	4.03	5.03
Matrix effect (%)	98	108	110	103	101

Accuracy of the chromatographic methods was checked by evaluating the recovery studies after spiking the known amount of each pesticide at low, medium, and high concentration levels as already described in section 2.5.

The percent (%) recoveries were achieved in ranges of 88.5–116.9% (Table 2) for all pesticides under investigation by using the proposed HPLC method. Such an achievement of percent recoveries justifies the validity and authenticity of the proposed technique for its intended applications. The recoveries obtained by the HPLC method complied with the standard (70–120%) set by European Union guidelines (Document No SANTE/12682/2019) (Commission, 2019). The results of repeatability and reproducibility in terms of % RSD (Table 2) show that the variations in the values for repeatability and reproducibility were lesser than 20% (in replicate of five) and thus comply with the standard (≤20%) set by European Union guidelines (Document No SANTE/12682/2019) (Commission, 2019). The lowest concentration in the calibration curve i.e., 20 μg/ml was spiked to the blank matrix of each vegetable and the matrix effect was reported in percentage recovery (Table 1). LOD and LOQ of each pesticide were determined by the proposed LC method (Table 1), lower values of detection limits showed a very pleasing capability of the method to detect and quantify the pesticides under study.

**TABLE 2:** Accuracy and precision studies.

Pesticide	Concentration (µg/ml), *n* = 5		Recovery (%) ± SD	Precision (% RSD)	
	Spiked	Found		Repeatability	Reproducibility
Azoxystrobin	20	17.98	89.9 ± 0.44	5.65	4.58
	60	58.49	97.5 ± 3.89	6.11	5.32
	100	104.44	104.4 ± 2.88	3.74	5.47
Chlorothalonil	20	19.58	97.9 ± 2.92	4.25	4.25
	60	64.49	107.5 ± 3.75	3.21	4.57
	100	104.61	104.6 ± 5.09	5.68	1.25
Difenoconazole	20	18.33	91.6 ± 3.25	6.87	4.58
	60	65.39	108.9 ± 3.71	2.58	3.87
	100	106.1	106.1 ± 2.08	1.68	4.25
Lufenuron	20	17.79	88.5 ± 1.02	3.68	5.67
	60	63.65	106.1 ± 2.14	4.57	5.47
	100	101.8	101.8 ± 2.51	5.11	5.89
Diafenthiuron	20	23.39	116.9 ± 1.11	5.32	4.58
	60	57.2	95.3 ± 1.03	1.56	4.89
	100	105.19	105.2 ± 2.66	2.89	6.32

### 3.3 Application to real samples

Pesticide residues in commonly used five vegetables samples were determined by HPLC and the results are tabulated in Table 3. Azoxystrobin is effective against early and late blight, as well as against powdery and downy mildew (Hou et al., 2016). According to analyzed results, azoxystrobin residue found in eggplant, capsicum, apple gourd, cauliflower, and sponge gourd was in a range between 0.01 and 0.43 mg/kg whereas their MRL values were found between 3.0 and 5.0 mg/kg for all the vegetables under current studies. The obtained results show that azoxystrobin residues in all selected vegetables are within their permissible limits designed by FAO/WHO.

**TABLE 3:** Pesticide residue in vegetables under study (mean ± SEM, *n* = 10).

Vegetable	Active substance	Concentration (mg residue/kg)	MRL (mg residue/kg)
Eggplant	Azoxystrobin	0.01 ± 0.005 (Min. 0.009, Max. 0.012)	3.0
	Chlorothalonil	0.55 ± 0.002 (Min. 0.42, Max. 0.58)	-
	Difenoconazole	0.50 ± 0.003 (Min. 0.41, Max. 0.53)	0.6
	Lufenuron	0.01 ± 0.001 (Min. 0.009, Max. 0.012)	-
	Diafenthiuron	0.001 ± 0.001 (Min. 0.0008, Max. 0.0011)	1.0
Capsicum	Azoxystrobin	0.02 ± 0.001 (Min. 0.007, Max. 0.022)	3.0
	Chlorothalonil	0.003 ± 0.0001 (Min. 0.0006, Max. 0.0034)	7.0
	Difenoconazole	0.05 ± 0.001 (Min. 0.008, Max. 0.058)	0.9
	Lufenuron	0.01 ± 0.001 (Min. 0.009, Max. 0.013)	0.8
	Diafenthiuron	0.01 ± 0.001 (Min. 0.008, Max. 0.014)	-
Apple gourd	Azoxystrobin	0.43 ± 0.002 (Min. 0.39, Max. 0.48)	3.0
	Chlorothalonil	0.44 ± 0.002 (Min. 0.38, Max. 0.52)	NA
	Difenoconazole	20.97 ± 0.22 (Min. 18.91, Max. 21.12)	0.6
	Lufenuron	0.33 ± 0.001 (Min. 0.19, Max. 0.42)	-
	Diafenthiuron	0.42 ± 0.002 (Min. 0.38, Max. 0.53)	-
Cauliflower	Azoxystrobin	0.23 ± 0.002 (Min. 0.19, Max. 0.29)	5.0
	Chlorothalonil	0.50 ± 0.001 (Min. 0.43, Max. 0.62)	5.0
	Difenoconazole	10.28 ± 0.67 (Min. 8.79, Max. 11.28)	2.0
	Lufenuron	0.07 ± 0.001 (Min. 0.008, Max. 0.015)	-
	Diafenthiuron	0.66 ± 0.002 (Min. 0.48, Max. 0.71)	0.02
Sponge gourd	Azoxystrobin	0.04 ± 0.001 (Min. 0.006, Max. 0.05)	3.0
	Chlorothalonil	1.83 ± 0.09 (Min. 1.28, Max. 1.95)	-
	Difenoconazole	40.33 ± 0.74 (Min. 38.85, Max. 41.15)	0.6
	Lufenuron	0.12 ± 0.001 (Min. 0.09, Max. 0.16)	-
	Diafenthiuron	1.34 ± 0.08 (Min. 1.18, Max. 1.55)	-

The azoxystrobin is rapidly metabolized in vegetables after 14 days. Therefore, a wait of 14 days must be done after the application of this class of chemicals before the harvesting and supply of these vegetables in the market to avoid the transfer and accumulation of these pesticides into humans. Chen et al. noted a decline in concentration of azoxystrobin residue in Chinese cabbage and kale from 4.10 to 0.63 mg/kg and from 13.21 to 0.10 mg/kg respectively within 18 days (Chen et al., 2004). Bagi et al., performed experiments to study the azoxystrobin residue in cucumber at the end of the pre-harvest interval (PHI) and found its residue level to 1.0 mg/kg which is below the maximum residue level (MRL) (Bagi et al., 2014).

Chlorothalonil like azoxystrobin is beneficial against early and late blight, and powdery and downy mildew therefore is excessively used (Hou et al., 2016). The chlorothalonil residue found in eggplant, capsicum, apple gourd, cauliflower, and sponge gourd was found in a range between 0.003 and 1.83 mg/kg while these values are in permissible limit defined by FAO/WHO (FAO JMPR Experts, 2002).

The study from China revealed that the azoxystrobin and chlorothalonil dissipated rapidly in cabbage with mean half-lives of 0.4 and 1.7 days respectively. In the cabbage, the terminal residues of azoxystrobin and chlorothalonil at the harvest time were below their maximum residue limit established by Codex Alimentarius Commission. Under the recommended dosage of azoxystrobin and chlorothalonil, the study suggested that the formulation of azoxystrobin and chlorothalonil (560 g/L, SC) may be safer for vegetables (Hou et al., 2016).

Difenoconazole residue in eggplant, capsicum, apple gourd, cauliflower, and sponge gourd was found between 0.50 and 40.33 mg/kg, whereas their MRL values were 0.6, 0.9, 0.6, 2.0, and 0.6 mg/kg respectively. These results show that difenoconazole residues in eggplant and capsicum are within the permissible limits designed by FAO-WHO, while that of apple gourd, cauliflower, and sponge gourd are above their permitted MRL value by FAO/WHO. Akhtar et al. studied the difenoconazole residue in guava and eggplant and round gourd vegetables. The results show that the concentration of difenoconazole in all commodities is above their maximum MRL value. In guava, the determined amounts of difenoconazole were 81.5 mg/kg while in eggplant and round gourd, these values were 5.62 mg/kg and 61.53 mg/kg respectively (Akhtar et al., 2018).

Lufenuron residue in eggplant, capsicum, apple gourd, cauliflower, and sponge gourd were obtained in ranges between 0.01 and 0.33 mg/kg. The results show that lufenuron residues in capsicum are within the permissible limits designed by FAO/WHO, whereas MRL for other vegetables is not defined. Malhat et al., conducted the field experiment using tomato crop to study the dissipation behavior of lufenuron. Results showed that lufenuron residues were lost with pre-harvest intervals of 7 days when its application was performed as per the instructions of manufacturer (Malhat et al., 2012). Hanafi et al., experimented to find out the lufenuron residue in green beans as well as in spring onions. The data showed that the initial deposition of lufenuron in onions was 0.17 mg/kg then it disappeared at slow rate but the residue level (0.06 mg/kg) after a week was still higher than the MRL (i.e. 0.02 mg/kg). However, lufenuron was not detectable in green beans (MRL = 0.02 mg/kg) in early days. It was shown that the deposition of respective pesticide depends on the different structure/nature of the two vegetables (Hanafi et al., 2010).

Diafenthiuron residue in selected vegetables was found in a range between 0.001 and 1.34 mg/kg and these results showed that diafenthiuron residues in eggplant are within their permissible limits designed by FAO-WHO, however the concentration in cauliflower was above the allowable limits (0.02 mg/kg). Another study of diafenthiuron residue in cauliflower from Pakistan revealed that the concentration found were beyond their MRL values set by FAO (FAO JMPR Experts, 2002; Panhwar and Sheikh, 2013).

It has also been reported that pesticides are repeatedly being sprayed, e.g. 15 times on cauliflower during the growth period. Therefore, it is important to monitor the pesticide load in the environment globally and preventive measures must be taken to reduce their use or at least to reduce the pesticides’ residue level to MRL to protect human beings from their harmful effects. Hence, analysis of pesticide residues in food (especially in vegetables) and their removal is gaining much importance. It has also been found that the vegetables are sprayed with different pesticides without providing any awareness on their respective concentrations and time intervals which can cause potential damages to human health and thus these concerns should be addressed to avoid unbearable losses to human beings. It is also a routine practice that farmers frequently apply pesticides to the vegetables and then supply the vegetables without even waiting for a day and thus these vegetables contain pesticide residues above their MRLs.

### 3.4 Human health risk assessment

EDI and THQ values were calculated for both adults and children by using the concentration of pesticides in respective vegetables. The results are summarized in Table 4.

**TABLE 4:** EDI and THQ values by consumption of pesticide-contaminated vegetables.

Pesticide	EDI (Adults/Children)	THQ (Adults/Children)	Health risk (Adults/Children)
Eggplant			
Azoxystrobin	4.73 × 10^–5^/7.18 × 10^–5^	2.4 × 10^–3^/3.59 × 10^–4^	No significant/No significant
Chlorothalonil	2.60 × 10^–3^/3.95 × 10^–3^	0.17/0.26	No significant/No significant
Difenoconazole	2.37 × 10^–3^/3.59 × 10^–3^	0.24/0.36	No significant/No significant
Lufenuron	4.73 × 10^–5^/7.18 × 10^–5^	2.36 × 10^–5^/3.59 × 10^–3^	No significant/No significant
Diafenthiuron	4.73 × 10^–6^/7.18 × 10^–6^	1.58 × 10^–2^/2.39 × 10^–3^	No significant/No significant
Capsicum			
Azoxystrobin	9.40 × 10^–5^/1.44 × 10^–4^	2.0 × 10^–4^/7.20 × 10^–4^	No significant/No significant
Chlorothalonil	1.42 × 10^–5^/2.15 × 10^–5^	9.47 × 10^–4^/1.43 × 10^–3^	No significant/No significant
Difenoconazole	2.36 × 10^–4^/3.59 × 10^–4^	2.36 × 10^–2^/3.59 × 10^–2^	No significant/No significant
Lufenuron	4.73 × 10^–5^/7.18 × 10^–5^	2.36 × 10^–3^/3.59 × 10^–3^	No significant/No significant
Diafenthiuron	4.73 × 10^–5^/7.18 × 10^–5^	1.58 × 10^–2^/2.39 × 10^–2^	No significant/No significant
Apple gourd			
Azoxystrobin	2.03 × 10–^3^/3.09 × 10^–3^	1.02 × 10^–2^/1.54 × 10^–2^	No significant/No significant
Chlorothalonil	2.08 × 10–^3^/3.16 × 10^–3^	0.14/0.10	No significant/No significant
Difenoconazole	9.92 × 10–^2^/1.51 × 10^–1^	9.92/15.1	Significant/Significant
Lufenuron	1.56 × 10–^3^/2.37 × 10^–3^	0.08/0.12	No significant/No significant
Diafenthiuron	1.97 × 10–^3^/3.02 × 10^–3^	0.66/1.01	No significant/Significant
Cauliflower			
Azoxystrobin	1.09 × 10–^3^/1.65 × 10^–3^	6.45 × 10^–3^/8.25 × 10^–3^	No significant/No significant
Chlorothalonil	2.37 × 10–^3^/3.59 × 10^–3^	0.16/0.24	No significant/No significant
Difenoconazole	4.86 × 10–^2^/7.38 × 10^–2^	4.86/7.38	Significant/Significant
Lufenuron	3.31 × 10–^3^/5.03 × 10^–4^	0.17/2.51 × 10^–2^	No significant/No significant
Diafenthiuron	3.12 × 10–^3^/4.74 × 10^–3^	1.04/1.58	Significant/Significant
Sponge gourd			
Azoxystrobin	1.89 × 10–^4^/2.87 × 10^–4^	9.45 × 10^–4^/1.44 × 10^–3^	No significant/No significant
Chlorothalonil	8.66 × 10–^3^/1.31 × 10^–2^	0.58/0.87	No significant/No significant
Difenoconazole	1.91 × 10–^1^/2.89 × 10^–1^	19.1/28.9	Significant/Significant
Lufenuron	5.68 × 10–^4^/8.62 × 10^–2^	2.84 × 10^–2^/4.31	No significant/Significant
Diafenthiuron	6.34 × 10–^3^/9.62 × 10^–3^	2.113/3.21	Significant/Significant

The highest EDI value was obtained for difenoconazole except for the eggplant where its value was found at the lower side out of the tested pesticides. EDI value of each pesticide in every vegetable was higher for children which ranged between 7.18 × 10 ^−5–^3.09 × 10^–3^, 2.15 × 10 ^−5–^1.31 × 10^–2^, 2.89 × 10 ^−1–^3.59 × 10^–4^, 7.18 × 10 _-_^−5^8.62 × 10^–2^, and 7.18 × 10 ^−6–^9.62 × 10^–3^ for azoxystrobin, chlorothalonil, difenoconazole, lufenuron, and diafenthiuron respectively. Data showed that the THQ values of difenoconazole and diafenthiuron (except for adults) were greater than one which indicates a significant impact on human health on consuming apple gourd, cauliflower, and sponge gourd. However, the THQ values were lesser than one for eggplant and capsicum which pose no significant risk on human health (Table 4). These studies reveal that children are more susceptible to the pesticides exposure on using the vegetables under investigation.

### 3.5 Advantages of the MSPD-HPLC method

Comparison of the proposed MSPD-HPLC method with literature reported methods is given in Table 5. The proposed method demonstrates some clear advantages over other methods. Conventional techniques such as solvent partitioning and liquid-liquid extraction by using various organic solvents like *n*-hexane, acetone, petroleum ether, acetonitrile, ethyl acetate, dichloromethane, and propanol has been employed for the extraction of pesticide residue present in different vegetable samples. But the MSPD shows an advantage over the other mentioned methods in terms of its performance as it is user friendly, more convenient, and much faster due to non-involvement of additional steps like centrifugation and filtration. In addition, only small volumes of solvents are utilized in this method due to more contact between sorbent phase and analyte, and it avoids the formation of troublesome emulsions as well. Due to above qualities, this technique is effective for extraction, isolation, clean-up, and pre-concentration of pesticide residue in complex matrices (Pashaei et al., 2020).

**TABLE 5:** Analytical features comparison of the proposed method with reported methods.

Vegetable	Technique (Extraction/Analysis)	LOD/LOQ (μg/kg)	Recovery (%)	Origin/References
Potato, Cabbage, Cauliflower, Tomato, Brinjal, Cucumber, Pea, Okra, Capsicum, Green chili, Coriander leaf, and Spinach	QuEChERS-DLLME/GC-MS	1.0–10/5.0–34	87–106	India Rai et al., 2016
Spinach, and Potato	QuEChERS-dSPE/GC-MS/MS	-/<10	70–120	Korea Lee et al., 2017
Cucumber	HS-SPME/GC-ECD	0.11/0.77	81–106	China Wu et al., 2016
Potato, Cabbage, Cauliflower, Carrot, Garlic, Broccoli, Leek, Celery, Ginger, Peas, Bean, and Lettuce	QuEChERS-dSPE/LC-MS/MS	0.1–1/0.5–5	77–110	India Narenderan et al., 2019
Potatoes	QuEChERS-dSPE/UHLC-MS/MS	0.4–1.0/2.0–5.0	81–113	China Chen et al., 2018
Potatoes	QuEChERS-dSPE/HPLC-DAD	0.9/2.7	86–90	Saudi Arabia Abd-Alrahman and Almaz, (2016)
Tomato, and Cucumber	SFE-MSPE/HPLC-UV	0.1/3.3	91–99	Iran Bagheri et al., 2016
Eggplant, Capsicum, Apple gourd, Cauliflower, and Sponge gourd	MSPD/HPLC-UV	1.12–1.61/3.73–5.36^a^	88.5–116.9	This study, Pakistan

^a^ Indicated the concentration in μg/mL.

A complete dispersion and disruption of sample in small sized particles is obtained in MSPD, which later provides a better surface for the extraction of the sample. In basic SPE methods, sample disruption is achieved in earlier steps because the sample should be in liquid form, and thus several components are discarded before the extraction. In addition, the sample is generally retained in the first few millimeters of the sorbent packed in the column in solid phase extractions, whereas in MSPD, the sample is dispersed throughout the column. Finally, the physical and chemical interactions of the components of the system are greater in MSPD than in SPE (Barker, 2000). In presented MSPD method, the florisil mixed with silica gel and sodium sulphate has become such a versatile material that it does not affect the sample matrix at all. It just adsorbs the molecules of organic nature and permits them to migrate through the sorbent surface, where polarity of eluent plays an important role in the recovery of the adsorbed material especially the pesticides. Just one precaution of activating the adsorbent was taken before using it for the recovery of pesticides from the vegetables’ samples.

## 4 Conclusion

The analysis of pesticide residues such as azoxystrobin, chlorothalonil, difenoconazole, lufenuron, and diafenthiuron in five commonly used vegetables’ samples (eggplant, capsicum, apple gourd, cauliflower, sponge gourd) was performed after successful extraction of respective pesticides by MSPD. All the pesticides under investigation were satisfactorily separated and determined with sufficient resolution in the presence of matrix, where matrix effect is vitally important in the development of LC method. The performance of the MSPD-HPLC analytical method was assessed for selectivity, linearity, accuracy, precision, matrix effect, LOD, LOQ. The obtained results revealed that the proposed method was quite efficient and adequately accurate and precise for its routine use in food quality control laboratories. EDI and HQ values were calculated by using the concentrations of pesticides determined in respective vegetables for both adults and children to assess their impact on health. The values of THQ suggested that the difenoconazole and diafenthiuron contaminated vegetables have significant effect on human health whereas children are more susceptible than adults to these pesticides exposure by consuming the vegetables under investigation. Although chemical pesticides are more reliable, effective and convenient to kill the pests, however these pesticides often cause food safety and environmental problems. To avoid their contamination and ensure food safety, alternative strategies like trap devices and pests killing lamps should be opted. Also the use of environment-friendly pesticides is recommended because they undergo photochemical degradation and transform into metabolites which are not hazardous to environment and human health. In a nut shell, a global monitoring of pesticide load in the environment is imperative and preventive measures must be taken to reduce their use and limit the pesticides residue level to MRL to protect human beings from their harmful effects. Hence, analysis of pesticide residues in food and their removal is an essential requirement. Moreover, strict and tighter regulations regarding pesticide residues in vegetables are highly recommended.

## Data Availability

All data generated or analysed during this study are included in this published article.
